# Long interspersed nuclear element 1 hypomethylation has novel prognostic value and potential utility in liquid biopsy for oral cavity cancer

**DOI:** 10.1186/s40364-020-00235-y

**Published:** 2020-10-23

**Authors:** Kiyoshi Misawa, Satoshi Yamada, Masato Mima, Takuya Nakagawa, Tomoya Kurokawa, Atsushi Imai, Daiki Mochizuki, Daichi Shinmura, Taiki Yamada, Junya Kita, Ryuji Ishikawa, Yuki Yamaguchi, Yuki Misawa, Takeharu Kanazawa, Hideya Kawasaki, Hiroyuki Mineta

**Affiliations:** 1 Department of Otorhinolaryngology /Head and Neck Surgery, 1-20-1 Handayama, Hamamatsu University School of Medicine, Hamamatsu, Shizuoka 431-3192 Japan; 2 Department of Otorhinolaryngology/Head and Neck Surgery, Graduate School of Medicine, Chiba University, Chiba, Japan; 3 Department of Otorhinolaryngology/Head and Neck Surgery, Jichi Medical University, Shimotsuke, Tochigi Japan; 4 Preeminent Medical Photonics Education and Research Center Institute for NanoSuit Research, Hamamatsu University School of Medicine, Hamamatsu, Japan

**Keywords:** LINE-1, 5-hmC, HNSCC, Disease-free survival, Oral cavity cancer

## Abstract

**Background:**

New biomarkers are urgently needed to improve personalized treatment approaches for head and neck squamous cell carcinoma (HNSCC). Global DNA hypomethylation has wide-ranging functions in multistep carcinogenesis, and the hypomethylation of long interspersed nucleotide element-1 (LINE-1) is related to increased retrotransposon activity and induced genome instability. However, little information is available regarding LINE-1 hypomethylation and its prognostic implications in HNSCC.

**Methods:**

In this study, we analyzed LINE-1 hypomethylation levels in a well-characterized dataset of 317 primary HNSCC tissues and 225 matched pairs of normal mucosa tissues, along with five oral cavity cancer (OCC) circulating tumor DNA (ctDNA) samples using quantitative real-time methylation and unmethylation PCR. The analysis was performed according to various clinical characteristics and prognostic implications.

**Results:**

The results demonstrated that LINE-1 hypomethylation levels were significantly higher in the HNSCC tissues than in corresponding normal tissues from the same individuals (*P* < 0.001). Univariate analysis revealed that high levels of LINE-1 hypomethylation were correlated with poor disease-free survival (DFS; log-rank test, *P* = 0.038), whereas multivariate analysis demonstrated that they were significant independent prognostic factor for DFS (hazard ratio: 2.10, 95% confidence interval: 1.02–4.36; *P* = 0.045). Moreover, samples with high LINE-1 hypomethylation levels exhibited the greatest decrease in 5-hydroxymethylcytosine (5-hmC) levels and increase in tumor-suppressor gene methylation index (*P* = 0.006 and *P* < 0.001, respectively). Further, ctDNA studies also showed that LINE-1 hypomethylation had high predictive ability in OCC.

**Conclusions:**

LINE-1 hypomethylation is associated with a higher risk of early OCC relapse, and is hence, a potential predictive biomarker for OCC. Furthermore, 5-hmC levels also exhibited predictive potential in OCC, based on their inverse correlation with LINE-1 hypomethylation levels. LINE-1 hypomethylation analysis, therefore, has applications in determining patient prognosis and real-time surveillance of disease recurrence, and could serve as an alternative method for OCC screening.

**Supplementary information:**

**Supplementary information** accompanies this paper at 10.1186/s40364-020-00235-y.

## Introduction

Head and neck squamous cell carcinoma (HNSCC) include cancers of the larynx, pharynx (naso-, oro-, and hypo-pharynx), and oral cavity, and constitute approximately 4% of all cancers worldwide, with more than 350,000 deaths annually [1]. The treatment strategy for patients with HNSCC is generally guided by tumor–node–metastasis (TNM) classification and clinical staging [2]. Current pre- and post-treatment surveillance for HNSCC patients involve clinical evaluation combined with flexible endoscopy and conventional imaging [3]. HNSCC is a heterogeneous disease, and although molecular studies have been used in distinguishing HPV-positive from HPV-negative HNSCC, validated molecular characterizations have not yet been established [4–6].

DNA hypomethylation is the initial and a major DNA methylation abnormality recognized in human cancers. The activity of the human retroelement long interspersed nucleotide element-1 (LINE-1) has persisted over time within the human genome, and its derepression is associated with genomic instability and tumor development [7]. Based on the data presented by Furlan et al., increased hypomethylation of LINE-1 was observed in formalin-fixed paraffin-embedded tissues of stage III–IVB oropharyngeal cancer patients who were at higher risk for early relapse [8]. Despite the accumulated knowledge about oropharyngeal cancer, hypomethylation of LINE-1 in HNSCC, including that in the larynx, hypopharynx, and oral cavity, is an area that remains to be explored.

In human cancers, DNA methylation alterations, such as global DNA hypomethylation and tumor suppressor gene (TSG) hypermethylation, lead to genomic instability or altered gene expression [9, 10]. The ten–eleven translocation protein family can convert 5-methylcytosine (5-mC) to 5-hydroxymethylcytosine (5-hmC) [11]. Herein, we provide the first evidence for the correlation among LINE-1 hypomethylation, 5-hmC, and TSG methylation in HNSCC, a concept that could help provide a better understanding of the role of genomic instability in HNSCC tumorigenesis.

The potential of circulating tumor DNA (ctDNA) as a prognostic biomarker has been recently established in several cancer management applications [12]. Dynamic qualitative and quantitative changes in ctDNAs at various cancer stages serve as a non-invasive diagnostic tool for identifying cancer relapses [13]. Nevertheless, the LINE-1 hypomethylation status in serum ctDNA from HNSCC patients has not yet been further investigated. Ultimately, to develop clinically valuable biomarkers, close collaboration between basic and clinical research is needed.

In the present study, we demonstrated that LINE-1 hypomethylation is associated with poor disease-free survival (DFS) and serves as a critical event in oral cavity cancer (OCC). Moreover, this study highlights that enhanced accumulation of 5-hmC levels is inversely correlated with global LINE-1 hypomethylation levels, providing a fundamental insight into the regulation of global hypomethylation in HNSCC. Finally, we report the results of a validation study performed to identify novel ctDNA-based epigenetic markers and document their ability to efficiently improve the diagnosis and prognosis in OCC.

## Patients and methods

### Tumor samples

Tumor samples were obtained during surgery from 317 primary HNSCC tissues and 225 normal tissues. All patients were treated at the Department of Otolaryngology, Hamamatsu University School of Medicine. All patients provided written informed consent under a protocol approved by our Institutional Review Board (Approval Date: October 2, 2015; Ethics Approval Code: 25–149). The average age of the patients was 65.8 years (range: 32–92 years). Among the patients, 261 were men and 56 were women. The primary tumor locations were the larynx (*n* = 64), hypopharynx (*n* = 87), oropharynx (*n* = 68), and oral cavity (*n* = 98). Clinical information was obtained from the clinical records.

### Bisulfite modification and quantitative real-time methylation and unmethylation PCR

Genomic DNA was extracted from fresh tissues using the QIAamp DNA Mini Kit (QIAGEN, Hilden, Germany). Sodium bisulfite conversion was performed using the MethylEasy Xceed Rapid DNA Bisulfite Modification Kit (TaKaRa, Tokyo, Japan). The primer sequences specific for LINE-1 and TSGs are shown in Table S1 (Additional file 1: Table S1) [14]. A standard curve for quantitative real-time methylation and unmethylation PCR (Q-MSP and Q-UMSP) was constructed by plotting five serially diluted standard solutions of EpiScope Methylated HCT116 gDNA (TaKaRa, Tokyo, Japan) and EpiScope® Unmethylated HCT116 DKO gDNA (TaKaRa, Tokyo, Japan). The normalized methylation value (NMV) was defined as follows: NMV = (TSGs-S/TSGs-FM)/(ACTB-S/ACTB-FM), where TSGs-S and TSGs-FM represent TSGs methylation level in sample and universally methylated DNA, respectively, and ACTB-S and ACTB-FM correspond to β-actin levels in samples and universally methylated DNA, respectively [15]. Known amounts of LINE-1 methylated and unmethylated DNA molecules were used to generate absolute standard curves (y = − 6.125 * log(x) + 26.54 and y = − 2.765 * log(x) + 21.19, respectively). The number of methylated or unmethylated LINE-1 sequence copies was extrapolated from the standard curves. The percentage of methylation was defined as the ratio between methylated molecules and the sum of methylated and unmethylated molecules [unmethylated copy number/ (methylated copy number + unmethylated copy number)].

### Enzyme-linked immunosorbent assay for 5-hmC quantification

The 5-hmC content of genomic DNA was determined using a Quest 5-hmC DNA enzyme-linked immunosorbent assay (ELISA) Kit (Zymo Research, Irvine, CA, USA). The assays were performed according to the manufacturer’s instructions, loading 100 ng of DNA per well and using 4 μg/mL of anti-5hmC polyclonal antibodies. Absorbance at 430 nm was measured using a SynergyH1 microplate reader and Gen5 software (BioTek, Winooski, VT, USA). The amount of 5-hmC was calculated as a percentage based on a standard curve generated using kit controls [16].

### Liquid biopsy

We tested LINE-1 hypomethylation levels in a validation study comprising five OCC patients using ctDNA obtained pre- and post-treatment. We isolated ctDNA from 4.0 mL plasma samples using affinity-based binding to magnetic beads as per manufacturer’s instructions (QIAamp MinElute ccfDNA Kit, QIAGEN, Hilden, Germany). Peripheral blood samples (10 mL each) were collected in cell-stabilizing tubes (Cell-Free DNA Collection Tube, Roche, CA, USA).

### Data and statistical analyses

The LINE-1 hypomethylation levels in 317 tumor samples as well as the overall patient characteristics were analyzed statistically. Receiver-operator characteristic (ROC) curve analyses were performed in 225 matched pair samples to compare LINE-1 hypomethylation levels between tumor and normal tissues. Kaplan-Meier curves and log-rank tests were used to estimate DFS in the different subgroups. Differences in the LINE-1 hypomethylation levels among the 317 patients according to their clinical information were examined using Fisher’s exact test. The prognostic value of methylation status was assessed using multivariate Cox proportional hazards analysis adjusting for age (≥ 75 versus < 75 years), sex, smoking status, alcohol intake, and tumor stage (I, II, and III versus IV). Spearman’s correlation analysis was used to identify whether 5-hmC levels or TSG methylation indexes (MI) affect LINE-1 hypomethylation. *P* values < 0.05 were considered statistically significant. Statistical analyses were performed using StatMate IV software (ATMS Co. Ltd., Tokyo, Japan) and the Stata/SE 13.0 system (Stata Corporation, TX, USA).

## Results

### Distribution of LINE-1 hypomethylation levels among HNSCC tumor samples

Tumor and normal mucosa LINE-1 hypomethylation levels in 225 matched pairs were examined using Q-MSP. The cancer tissues had significantly higher levels of LINE-1 hypomethylation (0.134 ± 0.151) than matched normal mucosa (0.023 ± 0.056; *P* < 0.001, by paired *t*-tests) (Additional file 2: Fig. S1A). Moreover, LINE-1 hypomethylation levels exhibited highly discriminative ROC curve profiles, which clearly distinguished HNSCC from normal mucosal tissues (area under the ROC [AUROC] = 0.8200). At the cutoff value of 0.029, the sensitivity was 72.89% and specificity was 84.89% (Youden index) (Additional file 2: Fig. S1B).

**Fig. 1:**
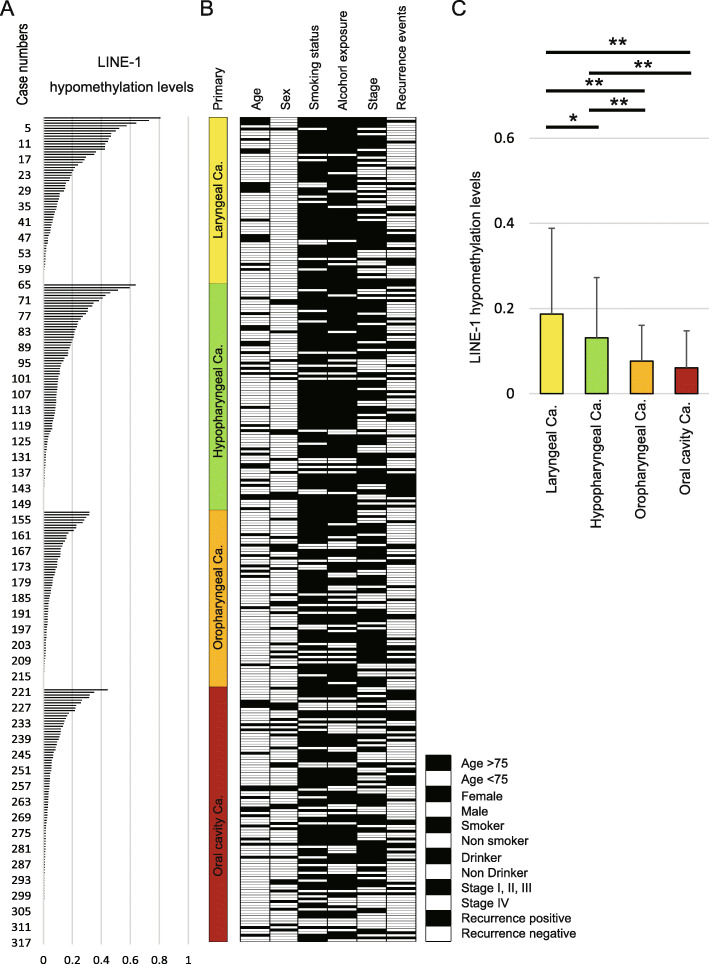
Distribution of LINE-1 hypomethylation levels and clinicopathological factors. **a**) Site-specific comparison of LINE-1 hypomethylation levels. Numbers in the left column represent the case numbers (labeled 1–317) and the bar graph illustrates LINE-1 hypomethylation levels among the 317 cases. **b** Filled and open boxes indicating clinicopathological discrimination. **c** Comparison of LINE-1 hypomethylation levels in various primary groups using Student’s *t*-tests. *P < 0.05, **P < 0.01

LINE-1 hypomethylation levels of the 317 tumor samples are presented in a horizontal bar graph (Fig. 1a). Clinicopathological classifications are shown in Fig. 1b. Site-specific analysis of LINE-1 hypomethylation levels are illustrated in Fig. 1c. These data indicate that LINE-1 hypomethylation levels were significantly higher in patients with laryngeal cancer (0.18 ± 0.20) than in patients with hypopharyngeal cancer (0.13 ± 0.14), oropharyngeal cancer (0.08 ± 0.08), and OCC (0.06 ± 0.09) (Fig. 1c).

### Association between LINE-1 hypomethylation levels and clinicopathological features

**Fig. 2:**
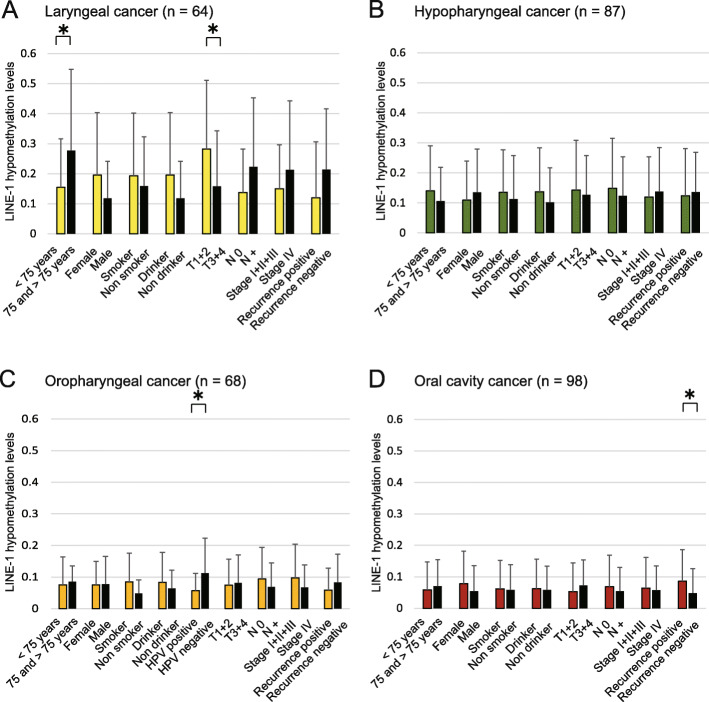
Association between LINE-1 hypomethylation levels and the selected clinical parameters. The mean LINE-1 hypomethylation levels for the various groups are compared using Student’s *t*-test. **a** Laryngeal cancer: statistically significant differences are found between LINE-1 hypomethylation levels and age as well as between LINE-1 hypomethylation levels and tumor size. **b** Hypopharyngeal cancer: no differences are noted for any of the clinical characteristics. **c** Oropharyngeal cancer: statistically significant differences are found between LINE-1 hypomethylation levels and HPV status (positive versus negative). **d** Oral cavity cancer: statistically significant differences are found between LINE-1 hypomethylation levels and recurrence events (positive versus negative). The mean and standard deviation are also indicated. *P < 0.05

No significant differences in LINE-1 hypomethylation levels were observed with respect to any clinical features (Additional file 3: Table S2). Site-specific analysis showed that LINE-1 hypomethylation levels in laryngeal cancer were significantly higher in the age groups of ≥75 years and in the T1 and T2 groups (Fig. 2a), but that there was no significant association between LINE-1 hypomethylation levels and clinicopathological characteristics in patients with hypopharyngeal cancer (Fig. 2b). Moreover, we found that LINE-1 hypomethylation levels were significantly higher in HPV-negative than in HPV-positive cases with oropharyngeal cancer (Fig. 2c). In the OCC patients, LINE-1 hypomethylation levels were significantly higher in recurrence-positive cases than in recurrence-negative cases (Fig. 2d).

### Association between LINE-1 hypomethylation levels and patient survival

**Fig. 3:**
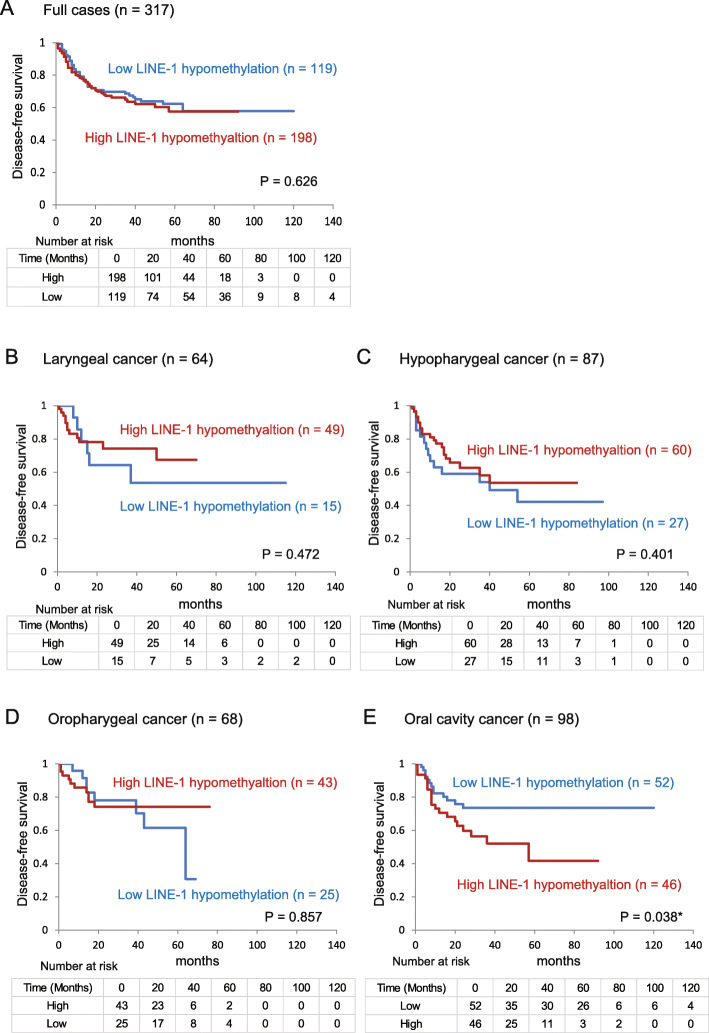
Kaplan–Meier survival curves according to LINE-1 hypomethylation levels. **a** Disease-free survival for all 317 head and neck squamous cell carcinoma (HNSCC) cases (*P* = 0.626). LINE-1 hypomethylation levels in patients with (**b**) laryngeal cancer (*n* = 64; *P* = 0.472), (**c**) hypopharyngeal cancer (*n* = 87; *P* = 0.401), (**d**) oropharyngeal cancer (*n* = 68; *P* = 0.857), and (**e**) oral cavity cancer (*n* = 98; *P* = 0.038). *P < 0.05.

Next, we confirmed the relationship between DFS and LINE-1 hypomethylation levels using Kaplan-Meier plots. Among the 317 HNSCC cases, no association between DFS and the high and low LINE-1 hypomethylation levels was observed (Fig. 3a). Moreover, no correlation was found between DFS and the high and low LINE-1 hypomethylation groups with laryngeal cancer (49 versus 15, *P* = 0.472; Fig. 3b), hypopharyngeal cancer (60 versus 27, *P* = 0.401; Fig. 3c), and oropharyngeal cancer (43 versus 25, *P* = 0.857; Fig. 3d). In patients with OCC, shorter DFS durations were observed in patients with high LINE-1 hypomethylation levels than those with low LINE-1 hypomethylation levels (46 versus 52, *P* = 0.038; Fig. 3e).

**Fig. 4:**
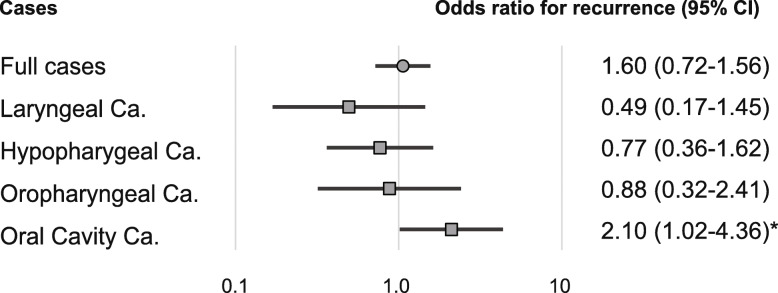
Risk of recurrence based on LINE-1 hypomethylation levels in tumors from various sites. Odds ratios for recurrence were determined using a Cox proportional hazards model adjusted for age (≥ 75 years versus < 75 years), sex, smoking status, alcohol intake, and stage (I–III versus IV). Thus, LINE-1 hypomethylation predicts poor outcome in oral cavity cancer patients. CI: confidence interval, *P < 0.05

In patients with OCC, the adjusted risk ratio for recurrence (RR) was 2.10 (95% confidence interval [CI]: 1.02–4.36, *P* = 0.045; Fig. 4).

### Comparison of LINE-1 hypomethylation levels with 5-hmC levels and TSG MI

**Fig. 5:**
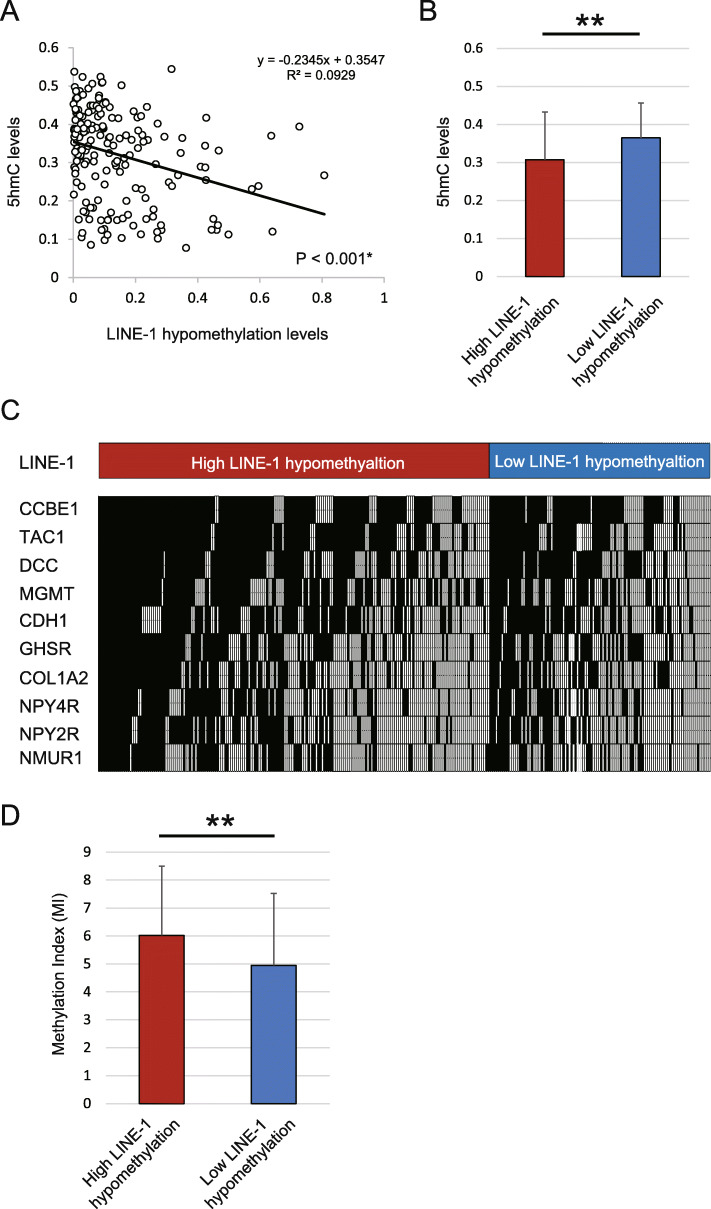
Comparison of 5-hmC levels and MI in 10 tumor suppressor genes with LINE-1 hypomethylation levels in primary HNSCC tissues. **a** Spearman rank correlations between 5-hmC levels and LINE-1 hypomethylation levels among 177 HNSCCs (R2 = 0.0929, *P* < 0.001). **b** Correlation between 5-hmC levels and LINE-1 hypomethylation levels in HNSCC patients (Student’s *t*-test; *P* = 0.006). **c** Distribution of LINE-1 hypomethylation levels and promoter methylation among the 10 tumor suppressor genes. Shaded boxes indicate the presence of methylation, whereas open boxes indicate the absence of methylation. **d** Correlation between MI and LINE-1 hypomethylation levels in HNSCC patients (*P* = 0.0003). The gene methylation rates for the different groups are compared using Student’s *t*-test. **P < 0.01

A significant negative correlation was observed between 5-hmC levels and LINE-1 hypomethylation levels (*R*^*2*^ = 0.0929; *P* < 0.001) in 177 HNSCC samples (Fig. 5a). The group with high LINE-1 hypomethylation levels had significantly lower levels of 5-hmC (0.31 ± 0.13) than group with low LINE-1 hypomethylation levels (0.36 ± 0.09, *P* = 0.006 by paired *t*-tests; Fig. 5b). The 10 TSGs were defined as the number of methylated genes in each sample (Fig. 5c). The mean differences in MI of the 10 TSGs were determined based on LINE-1 hypomethylation levels and are illustrated in Fig. 5d. In particular, MI was significantly higher in patients showing high LINE-1 hypomethylation levels (6.02 ± 2.47) than in those showing low LINE-1 hypomethylation levels (4.95 ± 2.58, *P <* 0.001; Fig. 5d). The 10 tumor suppressor genes were defined as the number of methylated genes in each sample (Additional file 4: Table S3).

### Validation of LINE-1 hypomethylation levels in ctDNA from OCC patients

**Fig. 6:**
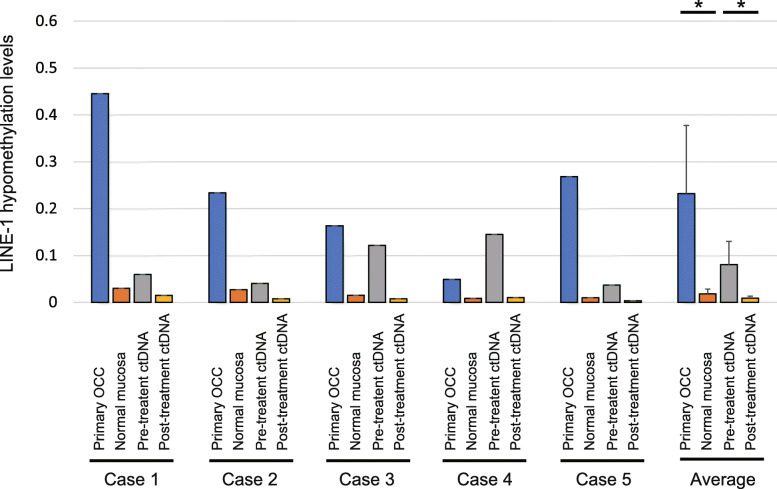
Validation analysis of LINE-1 hypomethylation levels in primary oral cavity cancer samples and paired ctDNA samples. Comparison of LINE-1 hypomethylation levels in five DNA specimens from primary samples, matched normal mucosa samples, and ctDNA isolated pre-treatment and post-treatment. *P < 0.05

Concordance was observed between the primary samples and matched pair normal mucosa samples (0.232 ± 0.146 versus 0.018 ± 0.010, *P* = 0.026; paired Student’s *t*-test). Moreover, we analyzed matched pair ctDNA for LINE-1 hypomethylation levels and found higher LINE-1 hypomethylation levels in pretreatment ctDNA than in post-treatment ctDNA (0.081 ± 0.049 versus 0.009 ± 0.004, *P* = 0.030; Fig. 6).

## Discussion

The current study elucidated the LINE-1 hypomethylation profiles in genomic DNA samples derived from the four anatomic sites in HNSCC and that higher LINE-1 hypomethylation levels were correlated with reduced survival in OCC. Although DNA methylation and its mechanism are well studied, DNA hypomethylation and demethylation are poorly understood in HNSCC [8, 17]. In a previous study, we showed that altered levels of 5-hmC were associated with tumorigenesis, and that lower 5-hmC levels were correlated with reduced survival [16]. However, the current study is the first to examine LINE-1 hypomethylation levels and 5-hmC levels in HNSCC. Additionally, our ctDNA findings showed that LINE-1 hypomethylation levels could aid in real-time disease surveillance and serve as an alternative method of screening for OCC.

Genome-wide hypomethylation, which is characterized by reduced methylation levels of LINE-1 and Alu, is often present in various malignancies [18]. DNA global hypomethylation in HNSCC is associated with smoking, alcohol consumption, and cancer stage [19]. Poage et al. reported a significant relationship of tumor site with LINE-1 hypomethylation levels (larynx > pharynx > oral cavity), but not with Alu and LUMA hypomethylation levels [17]. In uterine cervix carcinoma, there was a significant correlation between the degree of LINE-1 hypomethylation and progression from normal ectocervical mucosa to carcinoma in situ and invasive cancer [20]. Recent studies have also described that LINE-1 epigenetic aberrations may serve as biomarkers for novel screening and clinical management of esophageal squamous cell carcinoma [21, 22].

Global DNA hypomethylation has various important roles in multistep tumorigenesis [23]. Furthermore, 5-hmC is not simply an activating epigenetic mark, but also an intermediate in the active demethylation pathway, appearing to have complex roles in gene regulation [24]. Several studies have also shown that 5-hmC is an intermediate of DNA demethylation [25]. Immuno-based assays are the most commonly used, including ELISA, and these methods utilize available antibodies specific to 5-hmC to avoid cross-reactivity with 5-mC and unmethylated cytosine [16]. Moreover, 5-hmC depletion could significantly contribute to genomic instability and inaccurate chromosome segregation, perhaps explaining the correlation between low 5-hmC levels and cancer [26]. Furthermore, LINE-1 retrotransposition can lead to genomic instability and genetic heterogeneity in tumor-initiating cells [27]. Hence, both 5-hmC levels and LINE-1 hypomethylation may contribute to genomic instability in various human cancers, including HNSCC. As the first novel aspect of this study, we elucidated that a change in 5-hmC levels inversely correlated with LINE-1 hypomethylation in HNSCC.

LINE-1 hypomethylation outperforms existing clinical risk parameters as a prognostic biomarker. Several studies have investigated the relationship between ctDNA hypomethylation transitions in LINE-1 and clinical features in cancer patients. LINE-1 hypomethylation in ctDNA was linked to poor overall survival in diffuse large B cell lymphoma and to surrogate biomarkers for neuroblastoma tumor burden [28, 29]. As an additional evidence of its clinical benefit, LINE-1 hypomethylation is described as a blood biomarker to detect colorectal cancer, particularly in the early stages of the disease [30].

## Conclusion

Collectively, LINE-1 hypomethylation profiles in primary tumors may be used to identify patients with OCC who are at a higher risk of recurrence. Moreover, ctDNA analysis of LINE-1 hypomethylation levels in OCC has the potential to aid in patient prognosis and real-time disease surveillance. However, our data have not been able to provide evidence that LINE-1 hypomethylation and decrease in 5-hmC levels have an impact on global DNA hypomethylation in HNSCC. Future research is thus needed to address this issue, and to determine whether LINE-1 hypomethylation has distinct functional roles in OCC.

## Supplementary information

**Additional file 1: Table S1.** Q-MSP/UMSP Primer List

**Additional file 2: Fig. S1.** LINE-1 hypomethylation levels in matched pairs of HNSCC tissues and adjacent normal mucosal tissues. (A) Significant differences between cancer tissues and normal mucosal tissues are observed, as determined by Student’s *t*-test (*P* < 0.001). (B) The AUROC value for LINE-1 hypomethylation levels is 0.8200. At the cutoff value of 0.029, the sensitivity is 72.89%, and the specificity is 84.89%. AUROC: area under the receiver-operator characteristic.

**Additional file 3: Table S2.** The correlation between LINE-1 hypomethylation levels and clinical characteristics.

**Additional file 4: Table S3.** LINE-1 hypomethylation levels with the methylation of other ten genes.

## Data Availability

All data generated and analyzed during this study are included in this article and its supplementary information files.

## Abbreviations

HNSCC: Head and neck squamous cell carcinoma
LINE-1: Long interspersed nucleotide element-1;
ctDNA: Circulating tumor DNA
Q-MSP/UMSP: Quantitative real-time methylation and unmethylation PCR
5-hmC: 5-hydroxymethylcytosine
TSGs: Tumor-suppressor genes
TNM: Tumor–node–metastasis
TET: Ten-eleven translocation
5-mC: 5-methylcytosine
NMV: Normalized methylation value
ROC: Receiver-operator characteristic
