# Supplementary material for: Long interspersed nuclear element 1 hypomethylation has novel prognostic value and potential utility in liquid biopsy for oral cavity cancer
Source: Biomark Res. 2020 Oct 23;8:53. doi: 10.1186/s40364-020-00235-y (PMC7585304; doi:10.1186/s40364-020-00235-y)
Supplement: Supplementary file 1 — Additional file 1: Table S1. Q-MSP/UMSP Primer List [file 40364_2020_235_MOESM1_ESM.docx]

| **Table S1. Q-MSP/UMSP Primer List** | |  |
| --- | --- | --- |
| Gene | Forward primer 5’-3’ | Reverse primer 5’-3’ |
| ACTB | TGGTGATGGAGGAGGTTTAGAAGT | AACCAATAAAACCTACTCCTCCCTTAA |
| LINE-1 MSP | CGCGAGTCGAAGTAGGGC | ACCCGATTTTCCAAATACGACCG |
| LINE-1 UMSP | TGTGTGTGAGTTGAAGTAGGGT | ACCCAATTTTCCAAATACAACCATCA |
| CCBE1 | GTCGCGGAGGAGTAGGACGCTT | CTCGAAAACGACGACACCATC |
| TAC1 | GGCGGTTAATTAAATATTGAGCAGAAAGTCGC | AAATCCGAACGCGCTCTTTCG |
| DCC | TTGTTCGCGATTTTTGGTTTC | ACCGATTACTTAAAAATACGCG |
| MGMT | TTCGACGTTCGTAGGTTTTCGC | GCACTCTTCCGAAAACGAAACG |
| CDH1 | GTGGGCGGGTCGTTAGTTTC | ACCACAACCAATCAACGCGA |
| GHSR | TAGTATGTGGAACGCGACGT | AACTCGTCGCCCAACGAATC |
| COL1A2 | ACGGTAGTAGGAGGTTTCGG | CGCAAAACCCCTAAATCACCGACG |
| NPY4R | AGGTTGGGCGGGCGTAGGCGGGA | CGAAACAAAACCGCGCCTACTT |
| NPY2R | CGAGTGAGTGCGGTGTTTAGGCG | CGAACGAACAACCGAAACAATC |
| NMUR1 | GCGCGGGTTGGGGTCGTTGT | CGCACCTACCATACGACCCG |
