# Supplementary material for: Long interspersed nuclear element 1 hypomethylation has novel prognostic value and potential utility in liquid biopsy for oral cavity cancer
Source: Biomark Res. 2020 Oct 23;8:53. doi: 10.1186/s40364-020-00235-y (PMC7585304; doi:10.1186/s40364-020-00235-y)
Supplement: Supplementary file 2 — Additional file 2: Fig. S1. LINE-1 hypomethylation levels in matched pairs of HNSCC tissues and adjacent normal mucosal tissues. (A) Significant differences between cancer tissues and normal mucosal tissues are observed, as determined by Student’s t-test (P < 0.001). (B) The AUROC value for LINE-1 hypomethylation levels is 0.8200. At the cutoff value of 0.029, the sensitivity is 72.89%, and the specificity is 84.89%. AUROC: area under the receiver-operator characteristic. [file 40364_2020_235_MOESM2_ESM.docx]

| **Table S2 The correlation between LINE-1 hypomethylation levels and clinical characteristics.** | | | | | |
| --- | --- | --- | --- | --- | --- |
| Samples | | | LINE-1 hypomethylation levels | | |
| Characteristics | | Overall (%) | > 0.029 | < 0.029 | P |
| Age | < 75 | 252 (79.5%) | 154 | 98 |  |
|  | 75 and > 75 | 65 (20.5%) | 44 | 21 | 0.389 |
| Sex | female | 56 (17.7%) | 34 | 22 |  |
|  | male | 261 (82.3%) | 164 | 97 | 1 |
| Smoking status | smoker | 246 (77.6%) | 161 | 85 |  |
|  | non smoker | 71 (22.4%) | 37 | 34 | 1 |
| Alcohol exposure | drinker | 237 (74.8%) | 153 | 84 |  |
|  | non drinker | 80 (25.2%) | 45 | 35 | 1 |
| Tumor size | T1-2 | 143 (45.1%) | 82 | 61 |  |
|  | T3-4 | 174 (54.9%) | 116 | 58 | 0.103 |
| Lympho-node status | N0 | 119 (37.5%) | 77 | 42 |  |
|  | N+ | 198 (62.5%) | 121 | 77 | 0.551 |
| Stage | I, II, III | 125 (39.4%) | 77 | 48 |  |
|  | IV | 192 (60.6%) | 121 | 71 | 1 |
| Recurrence events | positive | 104 (32.8%) | 63 | 41 |  |
|  | negative | 213 (67.2%) | 135 | 78 | 1 |
| † Fisher’s exact probability test. | |  |  |  |  |
| * P < 0.05 |  |  |  |  |  |
