# Supplementary material for: Long interspersed nuclear element 1 hypomethylation has novel prognostic value and potential utility in liquid biopsy for oral cavity cancer
Source: Biomark Res. 2020 Oct 23;8:53. doi: 10.1186/s40364-020-00235-y (PMC7585304; doi:10.1186/s40364-020-00235-y)
Supplement: Supplementary file 3 — Additional file 3: Table S2. The correlation between LINE-1 hypomethylation levels and clinical characteristics. [file 40364_2020_235_MOESM3_ESM.docx]

| **Table S3. LINE-1 hypomethylation levels with the methylation of other ten genes.** | | | | |
| --- | --- | --- | --- | --- |
| Genes | Methylation status | LINE-1 hypomethylation levels | | |
|  |  | > 0.029 | < 0.029 | P-values χ |
| CCBE1 | Methylated | 150 | 76 |  |
|  | Unmethylated | 48 | 36 | 1 |
| TAC1 | Methylated | 159 | 60 |  |
|  | Unmethylated | 39 | 52 | < 0.001* |
| DCC | Methylated | 137 | 69 |  |
|  | Unmethylated | 61 | 43 | 1 |
| MGMT | Methylated | 136 | 55 |  |
|  | Unmethylated | 62 | 57 | < 0.001* |
| CDH1 | Methylated | 122 | 62 |  |
|  | Unmethylated | 76 | 50 | 1 |
| GHSR | Methylated | 108 | 55 |  |
|  | Unmethylated | 90 | 57 | 0.407 |
| COL1A2 | Methylated | 100 | 51 |  |
|  | Unmethylated | 98 | 61 | 0.41 |
| NPY4R | Methylated | 96 | 45 |  |
|  | Unmethylated | 102 | 67 | 0.192 |
| NPY2R | Methylated | 95 | 43 |  |
|  | Unmethylated | 103 | 69 | 0.122 |
| NMUR1 | Methylated | 89 | 38 |  |
|  | Unmethylated | 109 | 74 | 0.071 |
| χ Fisher’s exact probability test. | | | | |
| * P <0.05. | | | | |
