# Supplementary figures and images for: Long interspersed nuclear element 1 hypomethylation has novel prognostic value and potential utility in liquid biopsy for oral cavity cancer
Source: Biomark Res. 2020 Oct 23;8:53. doi: 10.1186/s40364-020-00235-y (PMC7585304; doi:10.1186/s40364-020-00235-y)

Figure S1 Kiyoshi Misawa

A

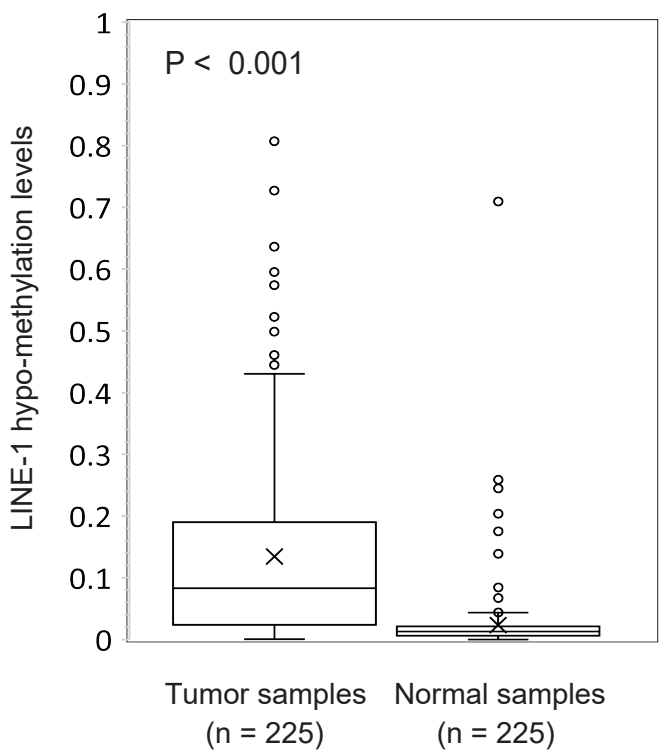

B

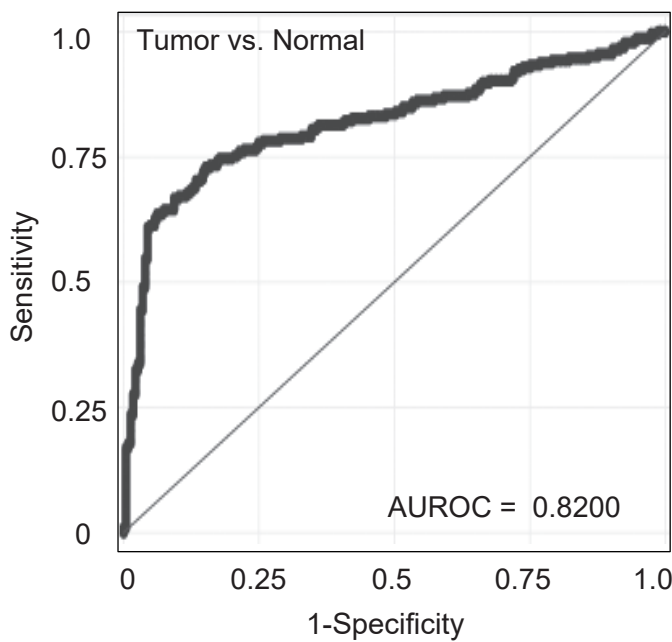

Supplement: Supplementary file 4 — Additional file 4: Table S3. LINE-1 hypomethylation levels with the methylation of other ten genes. [file 40364_2020_235_MOESM4_ESM.pdf]
